# Toposelective on-surface synthesis of curved π-extended oligomers based on bowl-shaped aromatics

**DOI:** 10.1039/d6sc01702a

**Published:** 2026-05-21

**Authors:** Marta Gómez-Gómez, Jorge Labella, Jonas Björk, Adriana E. Candia, Tomás Torres, Jorge Lobo-Checa

**Affiliations:** a Department of Organic Chemistry, Universidad Autónoma de Madrid Campus de Cantoblanco, C/Francisco Tomás y Valiente 7 28049 Madrid Spain jorge.labella@inv.uam.es tomas.torres@uam.es; b Materials Design Division, Department of Physics, Chemistry and Biology (IFM), Linköping University SE-58183 Linköping Sweden; c Laboratorio de Microscopias Avanzadas (LMA), Universidad de Zaragoza E-50018 Zaragoza Spain; d Instituto de Nanociencia y Materiales de Aragon (INMA), CSIC-Universidad de Zaragoza E-50009 Zaragoza Spain jorge.lobo@csic.es; e Institute for Advanced Research in Chemical Sciences (IAdChem), Universidad Autónoma de Madrid 28049 Madrid Spain; f IMDEA-Nanociencia, Campus de Cantoblanco 28049 Madrid Spain; g Departamento de Física de la Materia Condensada, Universidad de Zaragoza 50009 Zaragoza Spain

## Abstract

Curved π-extended systems based on bowl-shaped porphyrinoids are attractive because they exhibit unachievable properties in their planar counterparts. However, their synthesis typically yields oligomeric structures without topological control, a prerequisite for engineering materials with precise optoelectronic and physical properties. Here, we report a novel strategy to obtain exclusively *syn* topoisomers from *ortho*-dihalogenated subphthalocyanines (SubPcs) making use of on-surface synthesis methods. Using low-temperature scanning tunnelling microscopy (STM), supported by *ab initio* calculations, we show that the morphology and electronic structure of the products strongly depend on the halogen substituent in the precursor. Upon annealing up to 475 K, Br-substituted precursors yield covalently fused dimers and trimers sharing four- and six-membered rings, respectively, whereas F-substituted SubPcs form metal–organic chains mediated by F⋯Au and N⋯Au coordination. In contrast, Cl-substituted precursors, representing an intermediate case, require higher activation temperatures than their Br analogues, resulting in more irregular structures. In all cases, a single *syn* topology is observed that demonstrates the surface induced selectivity. In the covalent architectures, scanning tunnelling spectroscopy (STS) supported by Density Functional Theory (DFT) reveals that both the HOMO and LUMO are significantly perturbed upon oligomerization, resulting in modulation of the semiconducting energy gap consistent with effective π-delocalization. Altogether, these findings demonstrate that surface-assisted synthesis enables the toposelective construction of π-systems and highlight the decisive role of halogen selection in governing the formation of structurally and electronically defined architectures.

## Abstract 2

Toposelective on-surface synthesis of SubPc oligomers on Au(111) is enabled by bowl-down adsorption of *ortho*-dihalogenated precursors. The halogen substituent controls both the oligomerization outcome and the electronic properties.

## Graphical abstract

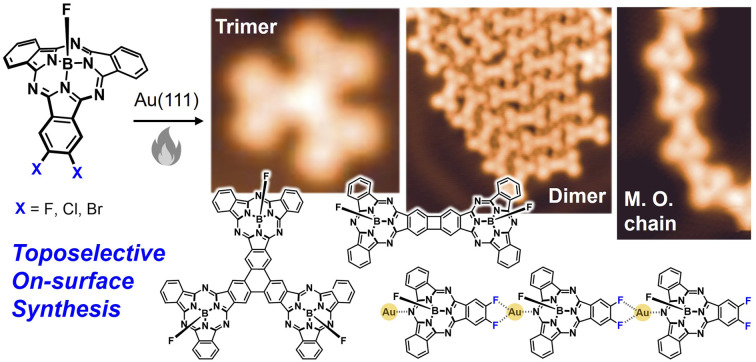

## Introduction

**Fig. 1:**
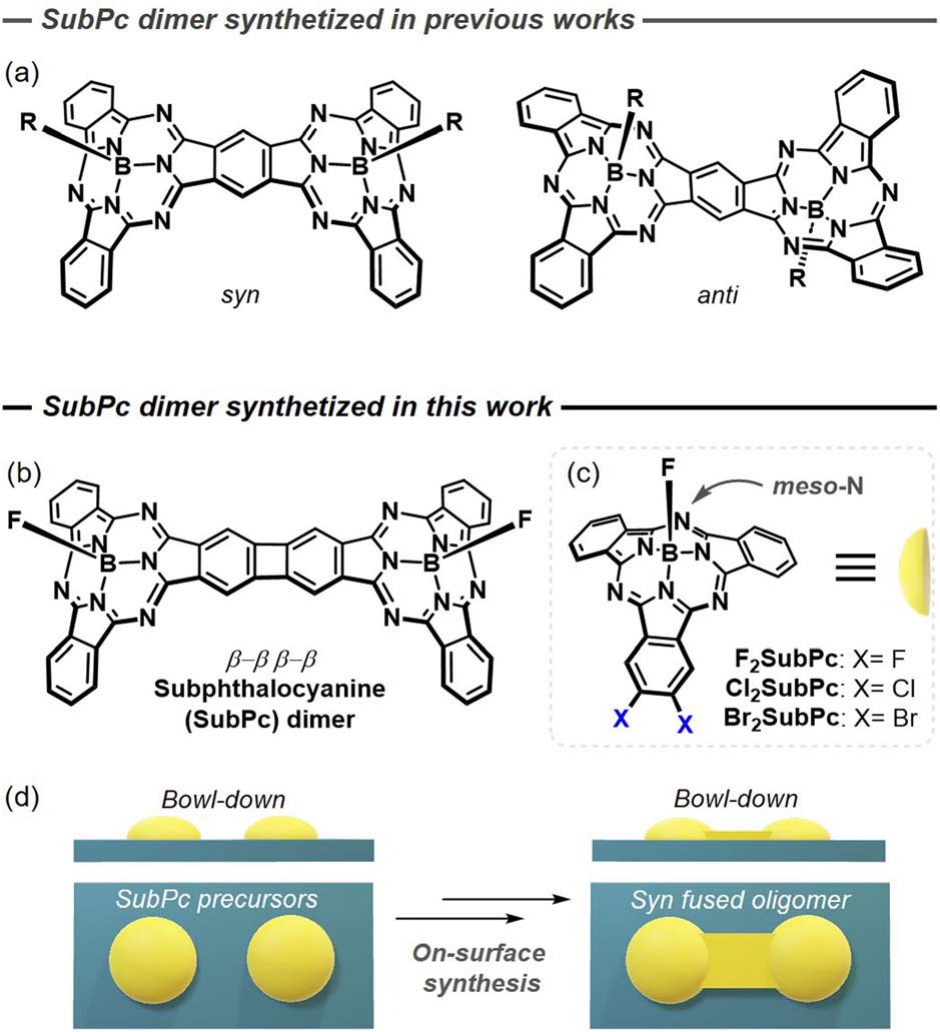
(a) Molecular structure of (*syn-* and *anti-*) SubPc fused dimers synthetized in solution in previous works. (b) Molecular structure of the *syn-*SubPc fused dimer prepared in this work. (c) Molecular structures of the compounds used in this work as precursors. (d) Illustration of the concept: on-surface catalyzed *toposelective* synthesis of dimers based on bowl-shaped porphyrinoids.

π-Extended oligomeric porphyrins have emerged as versatile platforms for engineering functional materials,^1^ as their tunable π-layout and π-conjugation enable precise control over charge transport,^2,3^ magnetic properties,^4,5^ and optical absorption.^6–9^ In this context, curved derivatives are attracting increasing attention because, while preserving efficient electronic delocalization, they exhibit distinctive features—such as enhanced solubility, strong fullerene affinity, and shape-assisted organization—that are not accessible to their planar counterparts.^10,11^ Curved π-extended porphyrinoids have been successfully accessed through several synthetic strategies, including the incorporation of seven- or eight-membered rings into the conjugated framework,^12–14^ the design of sterically congested architectures that enforce non-planarity,^2,15,16^ and metal-templated intramolecular C–C coupling reactions to construct cyclic π-arrays.^17,18^ An alternative approach still greatly unexplored is the use of ring-contracted porphyrinoids, which feature intrinsic bowl-shaped geometries.^19–21^ However, connecting derivatives that exhibit positive curvature under restricted rotational freedom—for example, upon fusion through a shared benzene ring—introduces an additional level of topological complexity, producing buckled architectures that may adopt either *syn* or *anti* arrangements with similar probability, reflecting comparable kinetic and thermodynamic profiles. This connection, as nicely illustrated by subphthalocyanine (SubPc) dimers sharing a central benzene ring (Fig. 1a), gives rise to isomers that differ in their π-topology, referred to as *topoisomers*.^22–24^

Fused SubPc dimers have shown promising applications as molecular materials due to their excellent supramolecular and optoelectronic properties. Notable is their use as non-fullerene acceptor in molecular photovoltaics^25^ and as photoactive materials in donor–acceptor systems.^26^ Despite their evident potential, the exploration of SubPc fused oligomers has been seriously hampered because: (i) their synthesis involves a non-efficient statistical cyclotrimerization of very specific phthalonitriles with challenging subsequent purification;^22^ (ii) the separation of *syn*- and *anti-topoisomers*, each with distinct properties (*e.g.*, solid-state packing, dipole moment, host-guest chemistry) and therefore different applications, is highly cumbersome;^27^ and (iii) the central shared aromatic unit must be benzene or pyrene, otherwise complex byproducts are produced during cyclotrimerization, or yields are reduced dramatically.^28^ On this basis, expanding the synthetic toolbox for SubPc-based oligomer synthesis would further enrich the exploration of their structural and functional potential. In this regard, the control over isomerism (*i.e.*, achieving *toposelectivity* in the reaction) is identified as a major obstacle and overcoming it would represent a significant advancement, magnified whenever the resulting products extend beyond dimers.

A sophisticated pathway to prepare π-extended porphyrin-based oligomers is on-surface synthesis on metal surfaces.^29–34^ SubPc molecules deposited on Au(111) surfaces have shown a bowl-down configuration, where the axial ligands point away from the surface while the π-concave backbone interacts with the substrate.^35,36^ This behaviour contrasts sharply with that of other bowl-shaped aromatics for which bowl-up, bowl-down, and even intermediate configurations have been observed.^37–39^ Thus, it is envisioned that the deposition of properly functionalized SubPcs on weakly interactive catalytic metal surfaces and subsequent on-surface coupling will lead to distinct *toposelective* formation of π-extended oligomers with a *syn* conformation (see Fig. 1b). To the best of our knowledge, *toposelectivity* remains yet unexplored in the chemistry of bowl-shaped aromatics.

In this work, we achieve the *toposelective* synthesis of SubPc-based oligomers by means of on-surface deposition of *ortho*-dihalogenated SubPcs (X_2_SubPc, where X = F, Cl and Br; see Fig. 1c) on Au(111). These isostructural precursors were selected because *ortho*-dihalogenated aromatic moieties are known to undergo oligomerization *via* [2 + 2] or [2 + 2 + 2] cycloadditions.^40,41^ In all cases, a F atom is introduced at the axial position (*i.e.*, the substituent bound to the boron atom) to prevent bowl-to-bowl inversion.^35^ By means of high-resolution low-temperature scanning tunnelling microscopy (STM), we find that both dehalogenation temperatures and structure formation are strongly halogen-dependent. Specifically, brominated precursors mainly yield π-fused dimers with scarce amounts of trimers, whereas fluorinated precursors form metal–organic chains linked by F⋯Au and N⋯Au interactions. Meanwhile, chlorinated precursors exhibit intermediate behavior, generating oligomers only at higher temperatures and leading to more irregular structures. Notably, all observed oligomers display *syn* topology, in agreement with theoretical simulations. Scanning tunnelling spectroscopy (STS), supported by Density Functional Theory (DFT) calculations, reveals that the frontier orbitals of the symmetric covalent oligomers are delocalized over the entire π-framework, and that the energy gap progressively narrows with increasing the number of SubPc units, both features consistent with efficient π-conjugation. Our results demonstrate not only that adsorption on a mildly interactive surface followed by on-surface synthesis is a powerful tool for achieving *toposelectivity*, but also that a wise selection of the substitutional halogen is essential to obtain the desired structure.

## Results and discussion

### Room temperature deposition arrangement

**Fig. 2:**
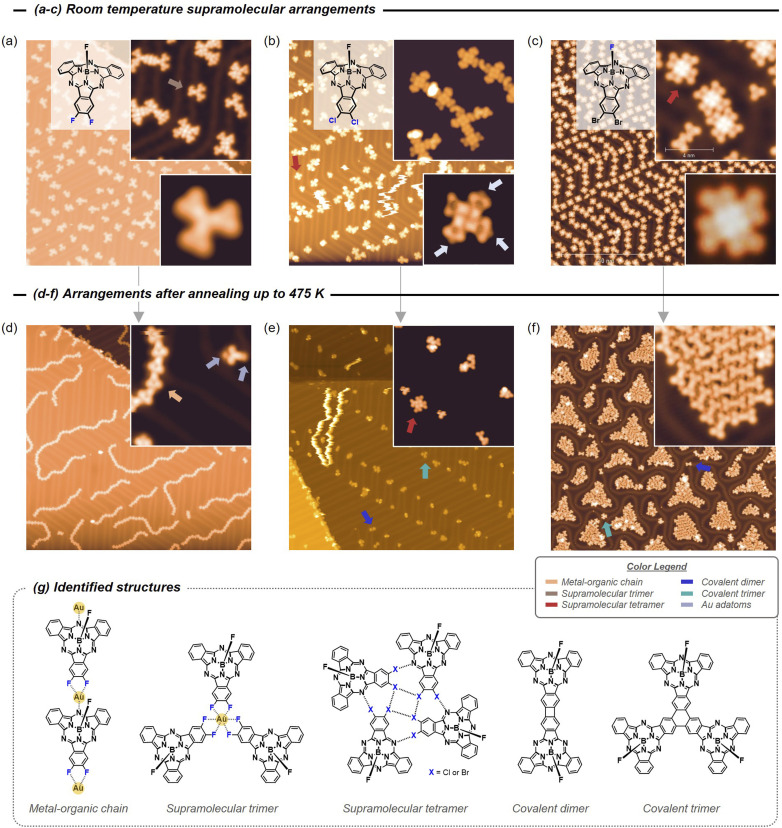
(a–c) Room temperature arrangements on the Au(111) of the three X_2_SubPc species. (a) The F_2_SubPc molecules form small structures mostly condensing around single-Au coordinated trimers. Halogen coordinated tetramers dominate in Cl_2_SubPc (b) and Br_2_SubPc (c) species. Cl_2_SubPc present a clear affinity for Au adatoms at *meso*-N atoms (marked by arrows in the bottom inset of (b)). (d–f) Arrangements of the three X_2_SubPc species on the Au(111) after annealing up to 475 K. (d) F_2_SubPc generates labile metal–organic chains. The inset shows a molecule with two Au atoms at opposite sites (indicated by arrows) after being dissected from a chain while scanning. (e) Cl_2_SubPc results in unreacted tetramers and covalent trimers, dimers or irregular structures. (f) Br_2_SubPc is fully reacted forming islands of packed covalent dimers separated by interstitial Br atoms, as well as single covalent dimers and trimers. (g) Schematic representations of the regular structures identified with the three X_2_SubPcs. The color legend for the arrows is included for clarity. STM image details: (a) *V*_B_ = 0.50 V, *I*_T_ = 50 pA, 108 × 108 nm^2^, inset top *V*_B_ = 0.01 V, *I*_T_ = 150 pA, 21.6 × 21.6 nm^2^; inset bottom *V*_B_ = 0.05 V, *I*_T_ = 30 pA, 4.3 × 4.3 nm^2^; (b) *V*_B_ = 0.25 V, *I*_T_ = 10 pA, 108 × 108 nm^2^, inset top *V*_B_ = 0.25 V, *I*_T_ = 10 pA, 16.2 × 16.2 nm^2^; inset bottom *V*_B_ = 0.005 V, *I*_T_ = 100 pA, 4.9 × 4.9 nm^2^; (c) *V*_B_ = −0.5 V, *I*_T_ = 100 pA, 108 × 108 nm^2^, inset top *V*_B_ = −0.01 V, *I*_T_ = 100 pA, 11.8 × 11.8 nm^2^; inset bottom *V*_B_ = −1.0 V, *I*_T_ = 170 pA, 3.8 × 3.8 nm^2^; (d) *V*_B_ = 0.50 V, *I*_T_ = 50 pA, 108 × 108 nm^2^, inset *V*_B_ = 0.01 V, *I*_T_ = 10 pA, 10.8 × 10.8 nm^2^; (e) *V*_B_ = 0.25 V, *I*_T_ = 50 pA, 108 × 108 nm^2^, inset *V*_B_ = 0.005 V, *I*_T_ = 100 pA, 21.6 × 21.6 nm^2^; (f) *V*_B_ = −0.50 V, *I*_T_ = 100 pA, 108 × 108 nm^2^, inset *V*_B_ = −0.3 V, *I*_T_ = 100 pA, 11.9 × 11.9 nm^2^.

Deposition of the three X_2_SubPc species on Au(111) was performed with the substrate kept at room temperature, where the monomers arrive intact and can easily diffuse on the surface. At this temperature, we found that the axial ligands point away from the surface and the molecules adopt the *bowl-down* configuration required for achieving *toposelectivity* (see Fig. 2a–c). Notably, we observed that the in-built halogen dictates the structures formed at room temperature: the F_2_SubPc molecules mainly condense in trimeric structures around a single Au native adatom, whereas Cl_2_SubPc and Br_2_SubPc form supramolecular tetramers without metal centers. These tetramers are stabilized by halogen⋯N and halogen···halogen interactions (see Fig. S5.5), as confirmed by DFT electrostatic potential (ESP) maps in Fig. S6.5, and exhibit chiral character following their clockwise or anticlockwise arrangements. In contrast, the metal–organic trimer arrangements displayed by F_2_SubPc originates from a strong affinity for native Au adatoms that bond *via* F⋯Au interactions already at room temperature (Fig. 2a). A similar binding motif has been recently reported in the hexafluorinated analogue (F_6_SubPc), yielding extended two-dimensional metal–organic frameworks.^36^ Interestingly, Cl_2_SubPc also exhibited affinity toward Au adatoms, albeit to a lesser extent and preferentially through *meso*-N coordination, enabling the condensation of four-fold units into short chains aligned along the herringbone reconstruction of the substrate (Fig. 2b). No significant interactions between SubPc molecules and Au adatoms were observed for Br_2_SubPc, whose tetramers remained structurally independent (Fig. 2c). These differences can be attributed to the charge distribution at the halogen positions, where the absence of electron-deficient σ-hole in F_2_SubPc precludes the dipole–dipole interactions, thereby preventing the tetramer formation. In contrast, tetramers derived from Cl_2_SubPc and Br_2_SubPc self-assembly do not host metal centers, which can be rationalized by the lack of physical space to accommodate them and the *s-*like character of the Au adatom orbitals being incompatible with the electronic environment dictated by the alternating halogen substituents (see Fig. S6.5).

### Post-annealing arrangement

To test the *toposelectivity* of the oligomeric products, we annealed the three systems to 475 K (see Fig. 2d–f). Despite their isostructural similarity, the halogen atoms have a deep effect on the final derivatives observed. In this regard, F_2_SubPc (Fig. 2d) generate labile chains where the molecules interact in a head-to-tail fashion. These wires are flexible since close tip-sample scanning conditions (*V*_B_ = 10 mV, *I*_T_ = 50 pA) can move them and even disrupt them if more severe scanning conditions are used (*V*_B_ = −5 mV, *I*_T_ = 150 pA). Such chain flexibility and the possibility to break them into single monomers (inset of Fig. 2d) evidence that at this temperature the F_2_SubPc molecules are not dehalogenated and maintain their initial integrity. Notably, Au adatoms were detected as circular features at two opposite ends of the molecules (between the two peripheral F atoms and the *meso*-N, greyish blue arrows at inset of Fig. 2d). Thus, these chains are stabilized by metal–organic (C–F)_2_⋯Au⋯N bonds (Fig. 2g). This behaviour contrasts sharply with that of F_6_SubPc, for which the (C–F)_2_⋯Au coordination density increases upon annealing on Au(111), while no *meso*-N⋯Au interactions are detected.^36^ We infer that this difference arises from the annealing process itself, which increases the surface concentration of mobile Au adatoms and generates additional coordination posibilities at the interface. Thus, F_2_SubPc open to coordination modes beyond F⋯Au interactions, namely *meso*-N⋯Au coordination, to satisfy the available adatom binding sites. In contrast, F_6_SubPc can coordinate a larger number of Au adatoms per molecule through its threefold dihalogenated scaffold, thereby stabilizing Au adatoms without requiring *meso*-N involvement, which is a less favorable coordination mode. This interpretation is supported by the observation that, upon room-temperature deposition, F_2_SubPc forms trimers predominantly *via* F⋯Au rather than *meso*-N⋯Au coordination. It should be noted that an increase of temperature (slightly above 500 K) leads to significant molecular desorption, although some irregular oligomers remain on the surface (see Fig. S5.1a).

In contrast, the Cl_2_SubPc molecules were partially dehalogenated after annealing to 475 K. Tetramers of unreacted molecules were residually identified on the surface, but small covalent structures (mostly irregular) were most abundant (Fig. 2e). Notably, we detected some symmetric trimeric and dimeric structures with *syn-toposelective* configuration, since their central axial ligands are found to be pointing into the vacuum. Note that no residual Cl atoms were detected on the surface and the Au adatom affinity persists as these are often found attached to the *meso*-N atoms of the molecules. Finally, when the temperature was increased above 500 K, the residual tetramers vanish from the surface replaced by covalent structures that at times take the shape of dimer and trimer units (Fig. S5.1b). However, we predominantly find irregular covalent oligomeric structures due to the higher temperature required for complete C–Cl bond activation concomitant with C–H bond activation at random molecular positions.

Lastly, annealing Br_2_SubPc to 475 K resulted in the dehalogenation of all molecules, fusing them into covalent dimers that aggregate into close packed islands (Fig. 2f). These dimers display symmetry and dimensions consistent with the expected SubPc–SubPc fusion through a shared four-membered ring. Interstitial Br atoms were identified between dimers, that are known to interact strongly with the surface to the point of lifting up the herringbone reconstruction.^42–44^ In addition, isolated symmetric trimers and dimers are seldomly spotted on the surface. Compared to the dimer, the trimers exhibited *C*_3_-symmetry, occurring after formation of a benzene ring at the central part of the structure (Fig. 2g). When increasing the temperature to 590 K, the residual Br atoms desorb, and the dimers can then interact and generate irregular covalent polymeric structures (Fig. S5.1c).

Except for the terminal halogen atoms, our monomers have been synthesized with identical structure, so we can use them to gauge the reactivity and product yield based on the choice of substitutional halogens. Our topographic images acquired at similar temperatures evidence that the structure formation and dehalogenation process is strongly element dependent on the Au(111) surface, while *syn-toposelectivity* is invariably obtained (see Fig. S5.4). Indeed, we found that covalent structures are scarce for F_2_SubPc with preferential monomer desorption, whereas metal–organic bonds are favored at intermediate temperatures. Contrarily, for Br_2_SubPc there is a strong predominance for symmetric dimer formation at 475 K (yield above 90%) that converts into disordered polymers at higher temperatures with practically no desorption. An intermediate case occurs for the Cl_2_SubPc, where around half of the precursors are desorbed close to 475 K before reacting forming covalent structures (mostly non-symmetric). Thus, the elemental halogen choice within the precursor strongly influences the structural selectivity of the oligomeric products. This follows the anticipated trend in reactivity; for example, C–F bonds are significantly more difficult to cleave than C–Br bonds.^45,46^

Another interesting aspect is that only Br is detected as a by-product after the on-surface synthesis process using Br_2_SubPcs precursors, while no halogens are identified on the surface for F_2_SubPc and Cl_2_SubPc. We attribute such difference to the fact that the C–F bonds of F_2_SubPc do not react on this surface before the precursors are desorbed, leaving just residual irregular structures that form *via* C–H bond activation. Contrarily, for Cl_2_SubPc, the high activation temperatures required for the Ullmann coupling prevent the stabilization of the Cl by-products on the Au(111) leaving a halogen-clean surface. Additionally, we observed that both the F_2_SubPc and Cl_2_SubPc species exhibit an affinity for the Au adatoms (stronger for the former) that is absent for Br_2_SubPc.

**Fig. 3:**
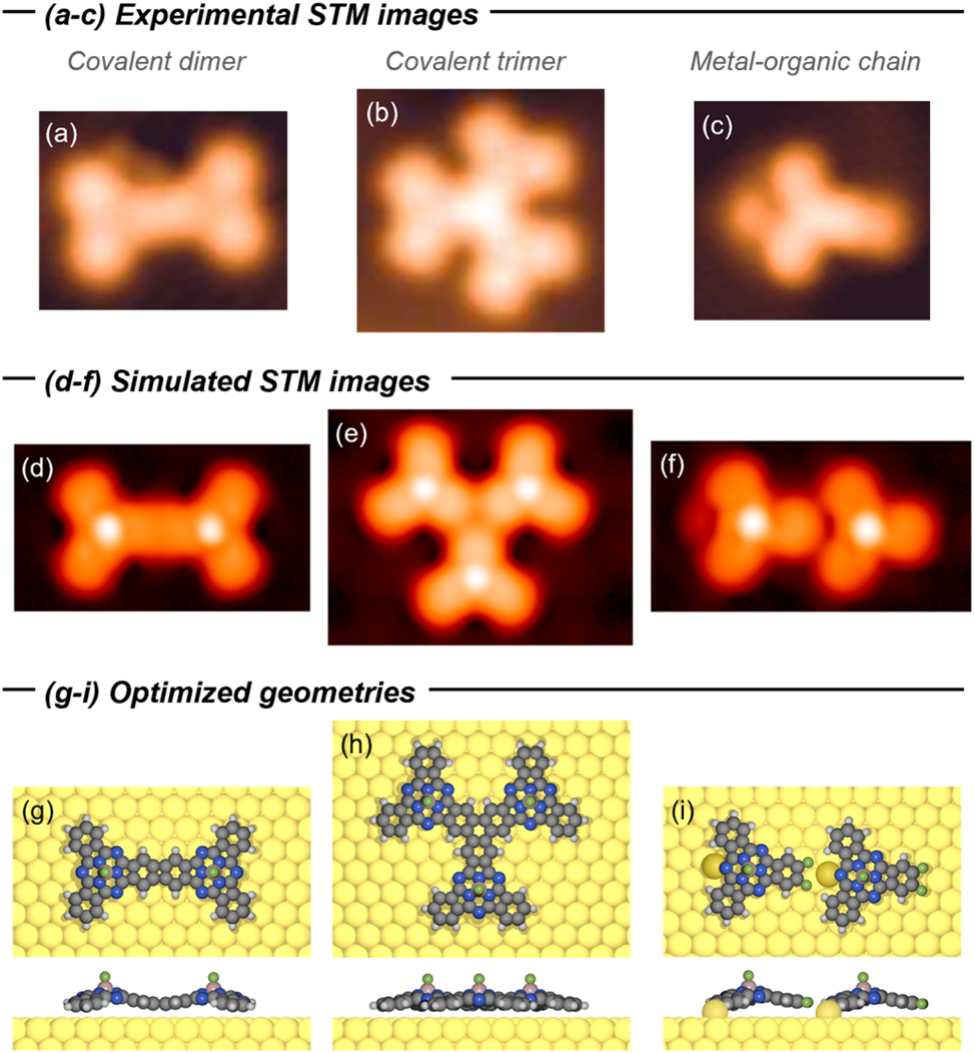
(a–c) Experimental and (d–f) simulated STM images, and (g–i) optimized geometries (top and lateral views) for the covalent dimer (a, d and g), covalent trimer (b, e and h) and metal–organic chain (c, f and i). Simulated STM images were obtained at constant LDOS at the Fermi level, analogous to constant-current imaging mode at low bias voltages. STM image details: (a) *V*_B_ = −0.30 V, *I*_T_ = 100 pA, 3.2 × 2.6 nm^2^; (b) *V*_B_ = −1.0 V, *I*_T_ = 200 pA, 3.5 × 3.5 nm^2^; (c) *V*_B_ = 10 mV, *I*_T_ = 10 pA, 2.8 × 2.6 nm^2^.

To gain further insight into the structure of the species generated upon annealing, DFT calculations were performed at employing the rev-vdW-DF2 non-local density functional.^47^ The optimized geometries, together with the comparison between simulated and experimental STM images, are shown in Fig. 3. The covalent dimers and trimers were modelled assuming fusion through four- and six-membered rings (*i.e.*, cyclobutadiene and benzene motifs), respectively. Gratifyingly, the simulated constant current STM images calculated close to the Fermi level show excellent agreement with the experimental data. Notably, both the dimer and trimer exhibit a corrugated topology after forming the new unsaturated rings, which arise from twofold or threefold C–C coupling (lateral views in Fig. 3g–i). This all-*syn* orientation of the SubPc units imposes a strong interaction with the Au(111) surface that compensates the intrinsic curvature upon monomer fusion. To model the metal–organic assemblies, a representative dimeric model of the chain motif was computed. Geometry optimization yields the coordination motif previously proposed, highlighting the unique ability of the curved SubPc precursor to coordinate Au adatoms either through the F atoms or the *meso*-N sites. Once again, the simulated images closely reproduce the experimental observations, clearly resolving the Au adatoms bridging the CF_2_ units and *meso*-N atoms along the chain. Importantly, this result provides direct evidence that the *meso*-N atoms of SubPcs can engage in metal coordination.

### Electronic characterization of covalent dimers and trimers

**Fig. 4:**
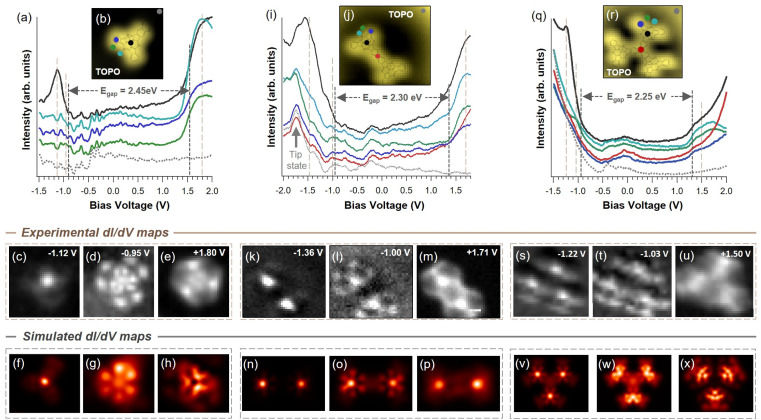
Electronic structure of a single molecule, a covalent dimer and a covalent trimer on Au(111). The data was extracted from three d*I*/d*V* grids. The top panels show position dependent spectra and constant current images (TOPO), whereas the middle and bottom panels exhibit the local density of states (LDOS): the middle panel correspond to experimental d*I*/d*V* isoenergetic maps, and the bottom panels, to the simulated constant-height d*I*/d*V* maps calculated. (a) to (h) correspond to a single molecule of (Br_2_SubPc) deposited at RT, (i) to (p) correspond to a covalent dimer after annealing Cl_2_SubPc, and (q) to (x) correspond to a covalent trimer after annealing Br_2_SubPc. The spectrum positions are indicated in the TOPO images by color code and the frontier orbital energy gaps are indicated with gray discontinuous vertical lines. The brown strips mark the energies of the bottom dI/dV maps, which show the spatial distribution of the different molecular orbitals. Experimental details. TOPO (b) *V*_B_ = −1.0 V, *I*_T_ = 179 pA, 2.7 × 2.7 nm^2^; (j) *V*_B_ = 0.05 V, *I*_T_ = 10 pA, 3.8 × 3.4 nm^2^; (r) *V*_B_ = −1.0 V, *I*_T_ = 200 pA, 2.9 × 2.7 nm^2^. d*I*/d*V* grid (a) to (e) 18 × 18 points with setpoint at −1.0 V, 170 pA, *V*_RMS_ = 9.5 mV, *f* = 817 Hz; (i) to (m) 38 × 33 points with setpoint at 0.20 V, 40 pA, *V*_RMS_ = 9.5 mV, *f* = 817 Hz; (q) to (u) 20 × 19 points with setpoint at −1.0 V, 200 pA, *V*_RMS_ = 20 mV, *f* = 817 Hz.

At this stage, we have established that all species derived from X_2_SubPc exhibit a *syn* topology. This common structural motif enabled the systematic investigation of the electronic properties of the symmetric fused dimers and trimers—species that have not been accessible *via* solution-phase synthesis—and their comparison with the monomer to elucidate the effect of π-extension (Fig. 4). The electronic characterization was performed by acquiring independent d*I*/d*V* grids, from which point spectra and conductance maps are extracted. Note that this turns out to be impracticable on the metal–organic F_2_SubPc structures given their conformational flexibility that impel the molecules to pivot around the metal center while acquiring the d*I*/d*V* datasets. Additionally, the measurement setpoint conditions were found to be decisive since it was possible to affect the integrity of the molecules by dissociating the axial ligand when using high positive bias (around 2 V) with large currents (1.2 nA) (see Fig. S5.2).

At the occupied region (negative bias voltages), all the measured species exhibits a dominant feature below −1.0 V, that is located at the axial ligand position (black curves in Fig. 4a,i and q). Indeed, the d*I*/d*V* maps around the maximum energy exhibits a single feature spatially situated at the SubPc B–F axial bond (Fig. 4c, k and s). However, this electronic state is not the Highest Occupied Molecular Orbital (HOMO), since a weaker state is found closer to the Fermi energy. In the monomer, the dI/dV map of this state (Fig. 4d) reveals a threefold symmetric distribution over the molecular backbone, involving both the benzene rings and the aromatic core, with six clearly resolved peripheral lobes (clover-like shape appearance). This spatial distribution, that fully correlates with the corresponding simulated state (Fig. 4g), is characteristic of the HOMO in monomeric SubPcs and is in excellent agreement with the DFT-calculated HOMO (see also Fig. S6.1). Upon formation of the dimer (Fig. 4l and o) and trimer (Fig. 4t and w), this molecular state becomes significantly perturbed, with a reduction in intensity at the central fusion region between the SubPc units (red curves in Fig. 4i and q). Consequently, the clover-shaped distribution characteristic of the monomer is modified, with the six peripheral lobes reduced to four per SubPc unit (see Fig. S6.2 and S6.3). In the unoccupied region (positive bias voltages), the first state observed in the monomer (Fig. 4e,h) exhibits its maximum intensity adjacent to the *meso*-N atoms (cyan curve in Fig. 4a), with linear features extending toward the peripheral benzene rings (green and blue curves in Fig. 4a). This threefold symmetric pattern appears to be complementary to the HOMO and is assigned to the Lowest Unoccupied Molecular Orbital (LUMO, see Fig. S6.1). Upon fusion into dimeric (Fig. 4m and p) and trimeric (Fig. 4u and x) structures, similar linear features were detected at the outer regions of the oligomers; however, near the central covalent fusion sites, these features overlap, leading to a slight modification of the orbital shape extending across the oligomer scaffold. This indicates efficient π-delocalization between the SubPc units in both the dimer and trimer upon covalent fusion, in agreement with the calculated HOMO and LUMO orbitals (see Fig. S6.2 and S6.3), which are consistent with the experimental and theoretical d*I*/d*V* maps.

Once the frontier orbitals (the HOMO and LUMO) have been identified, we quantify the energy gap of these three species. We find that the semiconducting character is progressively reduced as the number of SubPc units increases within the structure: 2.45 eV for the monomer, 2.30 eV for the dimer and 2.25 eV for the trimer (Fig. 4a, i and q). Such behavior, theoretically supported by calculations of the density of states for the three species (see Fig. S6.4), can be rationalized as the increment of the conjugation states that reduces the electron localization when the structure increases in size, thereby increasing the dispersion and bandwidth of the states.

It is important to mention that we observe that the Au affinity of the structures generated after annealing Cl_2_SubPc globally affects the electronic structure of the system. Indeed, for the trimer structure we detect a rigid energy shift of −220 mV when Au adatoms attach at the *meso*-N atoms of the molecule (Fig. S5.3). Importantly, this shift is opposite in sign to the documented halogen by-products existing after Ullmann coupling, which has been shown to rigidly shift the V_B_ and C_B_ onsets by +200 mV (to higher energies) in the case of zigzag covalent chains with adjacent Br adatoms.^48^ Such difference is expected, as the extrinsic doping of Au adatoms consists of an electron donating effect on the fused structures that is reversed upon halogen adatoms presence.

## Conclusions

In summary, we demonstrate that on-surface chemistry is a powerful tool to access curved π-extended systems under topological control. This is achieved using *ortho*-dihalogenated SubPcs, which tend to adopt a bowl-down configuration when deposited on Au(111). Owing to this defined adsorption orientation, subsequent on-surface coupling of the SubPc monomers exclusively produces a *syn* topology, in which all bowls are oriented toward the surface. Remarkably, this process strongly depends on the halogen substituent in the SubPc precursor: Br-substituted precursors undergo C–C coupling to yield predominantly dimers and, to a lesser extent, trimers, where the SubPc units are connected through four- and six-membered rings, respectively; Cl-substituted precursors exhibit C–X activation only at much higher temperatures, ultimately leading to desorption and the formation of irregular covalent structures; in contrast, F-substituted precursors preferentially coordinate to Au adatoms, forming metal–organic chains based on F⋯Au and *meso*-N⋯Au coordination. Our experimental and DFT datasets show that this covalent oligomerization perturbs both the HOMO and LUMO orbitals compared to the monomeric SubPc, leading to well-delocalized electronic states and a frontier orbital gap that decreases as the π-system expands in size and complexity. It is also worth noting that these studies clearly demonstrate that the *meso*-N atoms of SubPcs can engage in coordination chemistry. Moreover, this study represents a paradigmatic example of how a judicious choice of halogen substituent enables control over the final π-layout, which ultimately defines the properties and function of the material.

Overall, we anticipate that this work will stimulate the preparation of other curved π-extended oligomeric porphyrinoids on surfaces and guide future synthetic efforts in solution.

## Author contributions

J. L. and M. G.-G. synthesized and purified the molecules. A. E. C. and J. L.-C. conducted the STM/STS experiments and did the data analysis. J. L. and J. L.-C. performed the STM/STS data interpretation. J. B. performed the DFT calculations. J. L, M. G.-G., J. B. and J. L.-C. wrote the manuscript. J. L., T. T. and J. L.-C. conceived the project. All authors contributed to the revision and final discussion of the manuscript.

## Conflicts of interest

There are no conflicts to declare.

## Supplementary Material

SC-017-D6SC01702A-s001

## Data Availability

The data supporting the findings of this study are available within the article and its supplementry information (SI). Supplementary information: the following sections: instrumentation and materials, synthesis and compound data, NMR spectra, mass spectra, complementary on-surface studies, complementary computational studies. See DOI: https://doi.org/10.1039/d6sc01702a.
