# Supplementary material for: Toposelective on-surface synthesis of curved π-extended oligomers based on bowl-shaped aromatics
Source: Chem Sci. 2026 May 21;17(25):12609–16. doi: 10.1039/d6sc01702a (PMC13191369; doi:10.1039/d6sc01702a)
Supplement: SC-017-D6SC01702A-s001 [file SC-017-D6SC01702A-s001.pdf]

Supplementary Information (SI) for Chemical Science.
This journal is © The Royal Society of Chemistry 2026

Supplementary Information

Toposelective On-Surface Synthesis of Curved π-Extended Oligomers based on Bowl-Shaped Aromatics

Marta Gómez-Gómez,‡[a] Jorge Labella,‡†*[a] Jonas Björk,[b] Adriana E. Candia,†[c,d] Tomás Torres,*[a,e,f] Jorge Lobo-Checa*[c,d,g]

[a] Department of Organic Chemistry. Universidad Autónoma de Madrid, Campus de Cantoblanco, C/Francisco Tomás y Valiente 7, 28049 Madrid, Spain.

[b] Materials Design Division, Department of Physics, Chemistry and Biology (IFM), Linköping University, SE-58183 Linköping, Sweden

[c] Laboratorio de Microscopias Avanzadas (LMA), Universidad de Zaragoza, E-50018, Zaragoza, Spain

[d] Instituto de Nanociencia y Materiales de Aragon (INMA), CSIC-Universidad de Zaragoza, E-50009, Zaragoza, Spain

[e] Institute for Advanced Research in Chemical Sciences (IAdChem), Universidad Autónoma de Madrid, 28049 Madrid, Spain

[f] IMDEA-Nanociencia, Campus de Cantoblanco, 28049 Madrid, Spain

[g] Departamento de Física de la Materia Condensada, Universidad de Zaragoza, 50009, Zaragoza, Spain

† Present Addresses**:**
J.L.: Department of Molecular Engineering, Graduate School of Engineering, Kyoto University, Nishikyo-ku, Kyoto 615-8510, Japan.
A.E.C.: Instituto de Física del Litoral, Consejo Nacional de Investigaciones Científicas y Técnicas, Universidad Nacional del Litoral (IFIS-Litoral, CONICET-UNL), 3000, Santa Fe, Argentina

‡ M.G.-G. and J.L. contributed equally to this work.

e-mail: tomas.torres@uam.es, jorge.labella@inv.uam.es, jorge.lobo@csic.es

<u>Table of content</u>

1. Instrumentation and Materials 2
2. Synthesis and Compound Data 3
3. NMR Spectra 5
4. Mass Spectra 8
5. Complementary On-surface Studies 10
6. Complementary Computational Studies 13
7. Supporting References 17

Abbreviations

APCI-Q-TOF: Atmospheric-Pressure Chemical Ionization Quadrupole Time-of-Flight; DCM: Dichloromethane; DIPEA: *N,N*-diisopropylethylamine; HOMO: Highest Occupied Molecular Orbital; HR-MS: High Resolution Mass Spectrometry; LUMO: Lowest Unoccupied Molecular Orbital; MALDI-TOF: Matrix-Assisted Laser Desorption/Ionization-Time of Flight; Mp: Melting point; NMR: Nuclear Magnetic Resonance; ppm: part per million; STM: Scanning Tunneling Microscopy; SubPc: Subphthalocyanine; THF: Tetrahydrofuran; TLC: Thin Layer Chromatography; UV-vis: Ultraviolet-visible.

1. Instrumentation and Materials

The monitoring of the reactions has been carried out by thin layer chromatography (TLC), employing aluminum sheets coated with silica gel type 60 F254 (0.2 mm thick, Merck). The analysis of the TLCs was carried out with an UV lamp of 254 and 365 nm. Purification and separation of the synthesized products were performed by normal-phase column chromatography, using silica-gel 60 (230–400 mesh, 0.040–0.063 mm, Merck) as the stationary phase. Eluents along with the relative ratio in the case of solvent mixtures are indicated for each particular case.

Nuclear magnetic resonance spectra (^{1}H-, ^{13}C-,^{11}B-, ^{19}F-NMR) were recorded on a Bruker AV-300 or Bruker DRX-500 spectrometers. Deuterated solvent employed in each case is indicated in brackets, and its residual peak was used to calibrate the spectra using literature reference δ ppm values.[1] All the experiments were recorded at room temperature. Multiplicity was indicated using the following abbreviations: s (singlet), bs (broad singlet), d (doublet), dd (doublet of doublets), t (triplet), q (quartet), m (multiplet).

High-resolution mass spectra (HR-MS) were recorded in the Interdepartmental Investigation Service of UAM, employing matrix-assisted laser desorption/ionization time-of-flight (MALDI-TOF) using a Bruker-Ultraflex-III spectrometer with a Nd:YAG laser operating at 355 nm or atmospheric-pressure chemical ionization quadrupole time-of-flight (APCI-Q-TOF) using a Bruker-MAXIS-II spectrometer. The matrixes and internal references employed are indicated for each spectrum. Mass spectrometry data are expressed in m/z units.

Ultraviolet and visible (UV-vis) spectra were recorded using solvents in the spectroscopic grade in the Organic Chemistry Department of UAM employing a JASCO-V760 spectrophotometer.

All reactions dealing with air or moisture sensitive compounds were carried out by standard Schlenk techniques in a dry reaction vessel under argon. Chemicals were purchased from commercial suppliers and used without further purification. Dry solvents were purchased from commercial suppliers in anhydrous grade or thoroughly dried before use, employing standard methods. Solid, hygroscopic reagents were dried in a vacuum oven before use.

The synthesis and characterization of **4,5-dibromophthalonitrile**,[2] has been previously reported.

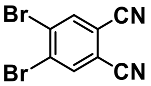

4,5-dibromophthalonitrile

2. Synthesis and Compound Data

General procedure for synthesizing X_2SubPcs

In a 50 mL two-necked round-bottomed flask, equipped with a condenser, magnetic stirrer and rubber seal, a 1.0 M solution of BCl_3 in *p*-xylene (10.2 mL) was added over the corresponding mixture of phthalonitriles (6.8 mmol, 0.9 g of phthalonitrile and 3.4 mmol of the corresponding 4,5-dihalogenatedphthalonitrile: 4,5-difluorophthalonitrile for **F_2SubPc**, 4,5-dichlorophthalonitrile for **Cl_2SubPc**, and 4,5-dibromophthalonitrile for **Br_2SubPc**) under argon atmosphere. The reaction mixture was stirred at 137 ºC for 30 min. The purple solution was allowed to cool to room temperature. Then, it was flushed with argon to remove the excess of BCl_3. After removal of the solvent, the 4-*tert*-butylphenol (17 mmol, 2.6 g), DIPEA (*N*,*N*-diisopropylethylamine; 3.4 mmol, 0.6 mL) and dry toluene (10.2 mL) were added over the crude under argon atmosphere. The new reaction mixture was stirred at 115 °C for 16 h. The dark purple reaction slurry was dissolved in a toluene/AcOEt 10:1 mixture and passed through a short silica plug. The solvent was removed by vacuum distillation and the resulting dark solid was purified by column chromatography on silica gel (the corresponding eluent is indicated in each case), in order to isolate the desired X_2SubPc bearing axial 4-*tert*-butylphenoxy-, which is obtained as a bright purple solid upon precipitation in a DCM/MeOH mixture. Then, this product (0.12 mmol), $BF_3·OEt_2$ (6.1 mmol, 0.75 mL) and dry toluene (1 mL) are added on a 10 mL Schlenk flask equipped with a magnetic stirrer, under argon atmosphere. The reaction mixture was stirred at 115 ºC for 4 h. Then, THF (1 mL) is added over the reaction crude. After removal of the solvents, the final product is obtained as a purple solid upon precipitation in DCM/MeOH.

F_2SubPc:

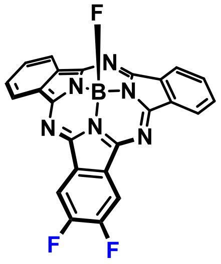

Eluent: toluene. Yield: 7 % (107 mg).

^{1}H-NMR (500 MHz, THF-d$_8$): δ (ppm) = 8.86−8.81 (m, 4H), 8.75 (t, $^3J_{H\text{-}F}$ = 8.6 Hz, 2H), 7.99−7.95 (m, 4H); **^{13}C-NMR** (126 MHz, THF-d$_8$) δ (ppm) = 154.3, 154.2, 153.8, 152.3, 152.2, 151.8 (d, $^1J_{C\text{-}F}$ = 248.2 Hz), 132.4 (d, $^2J_{C\text{-}F}$ = 27.2 Hz), 131.3, 131.2, 128.3, 123.1, 122.9; **^{11}B-NMR** (160 MHz, THF-d$_8$) δ (ppm) = −14.0 (d, J = 30.3 Hz, 1B; B−F); **^{19}F-NMR** (471 MHz, THF-d$_8$): δ (ppm) = −133.2 (s, 2F), −158.0 (q, J = 29.5 Hz, 1F; B−F); **UV-vis** (THF): λ (nm) (log ε/dm^3 mol^{-1} cm^{-1}) = 557 (4.9), 542 (sh), 515 (sh), 305 (4.6); **HR-MS** (MALDI-TOF): Calculated for $C_{24}H_{10}BF_3N_6$: 450.1011, found: 450.1006; **Mp** > 250 ºC.

Cl_2SubPc:

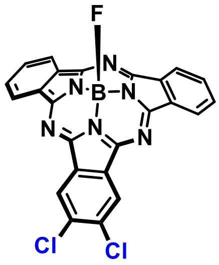

Eluent: toluene/AcOEt 20:1. Yield: 8 % (131 mg).

^{1}H-NMR (500 MHz, THF-d_8): δ (ppm) = 9.03 (s, 2H), 8.86−8.82 (m, 4H), 8.00−7.96 (m, 4H); **^{13}C-NMR** (126 MHz, THF-d_8) δ (ppm) = 154.2, 153.0, 150.1, 134.5, 132.6, 132.3, 131.5, 131.3, 124.5, 123.2, 123.0; **^{11}B-NMR** (160 MHz, acetone-d_6) δ (ppm) = −13.7 (d, J = 29.3 Hz, 1B; B−F); **^{19}F-NMR** (471 MHz, acetone-d_6): δ (ppm) = −156.3 (q, J = 28.6 Hz, 1F; B−F); **UV-vis** (THF): λ (nm) (log ε/dm^3 mol^{-1} cm^{-1}) = 567 (4.6), 557 (sh), 523 (sh), 307 (4.4); **HR-MS** (APCI-Q-TOF): Calculated for $C_{24}H_{10}BCl_2FN_6$+H: 483.0494, found: 483.0482; **Mp** > 250 ºC.

Br_2SubPc:

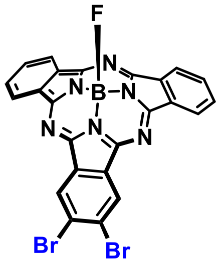

Eluent: toluene/AcOEt 20:1. Yield: 5 % (97 mg).

^{1}H-NMR (300 MHz, THF-d_8): δ (ppm) = 9.18 (s, 2H), 8.86−8.82 (m, 4H), 8.00−7.96 (m, 4H); **^{13}C-NMR** (126 MHz, THF-d_8) δ (ppm) = 153.0, 151.9, 148.8, 131.4, 131.2, 130.3, 130.1, 126.5, 125.5, 122.0, 121.9; **^{11}B-NMR** (160 MHz, THF-d_8) δ (ppm) = −13.9 (d, J = 29.8 Hz, 1B; B−F); **^{19}F-NMR** (471 MHz, THF-d_8): δ (ppm) = −157.9 (q, J = 29.5 Hz, 1F; B−F); **UV-vis** (THF): λ (nm) (log ε/dm^3 mol^{-1} cm^{-1}) = 569 (4.7), 557 (sh), 527 (sh), 309 (4.4); **HR-MS** (APCI-Q-TOF): Calculated for $C_{24}H_{10}BBr_2FN_6$+H: 572.9469, found: 572.9457; **Mp** > 250 ºC.

3. NMR Spectra

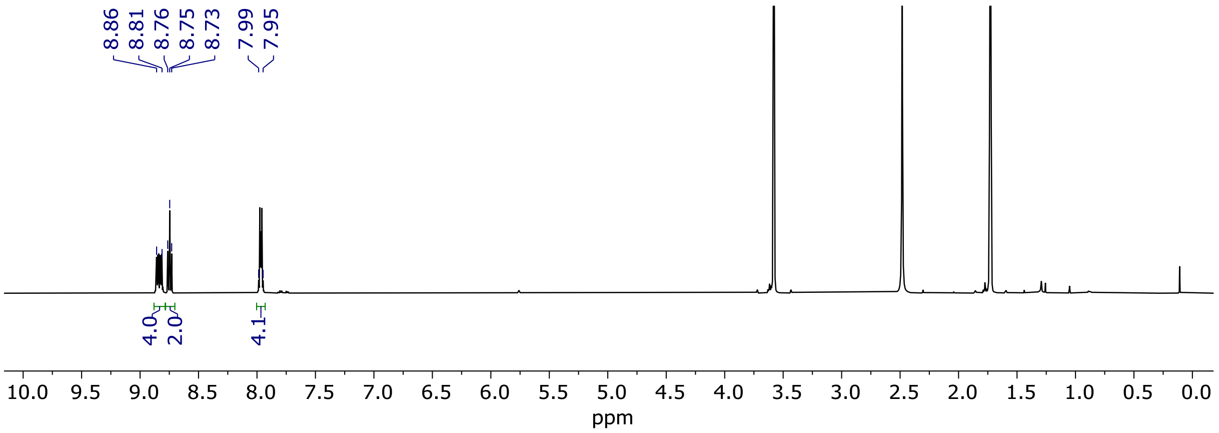

Figure S3.1. ^{1}H-NMR spectrum (THF-d_8) of **F_2SubPc.**

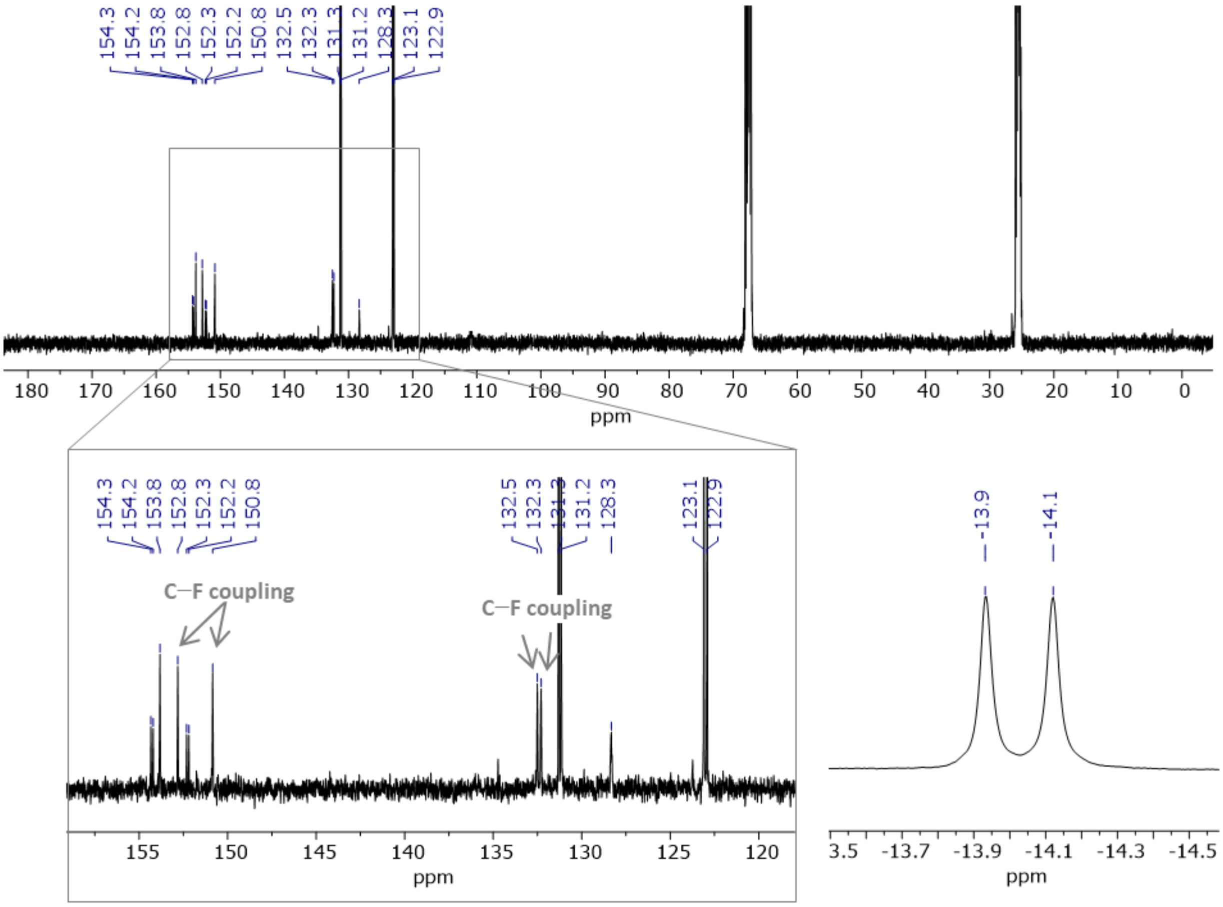

Figure S3.2. ^{13}C-NMR (top) and ^{11}B-NMR (bottom) spectra (THF-d_8) of **F_2SubPc**.

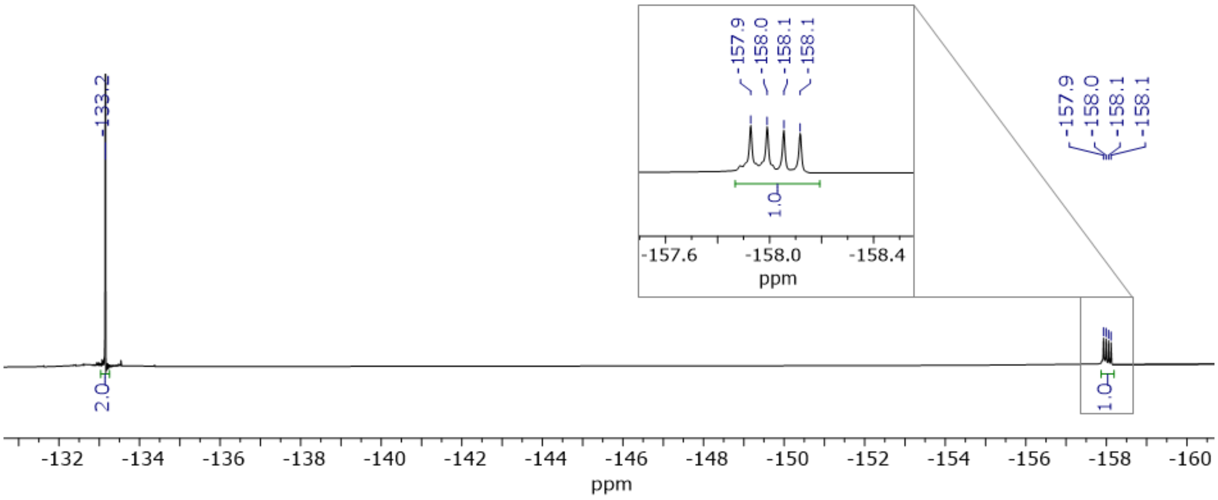

Figure S3.3. ^{19}F-NMR spectrum (THF-d_8) of **F_2SubPc**.

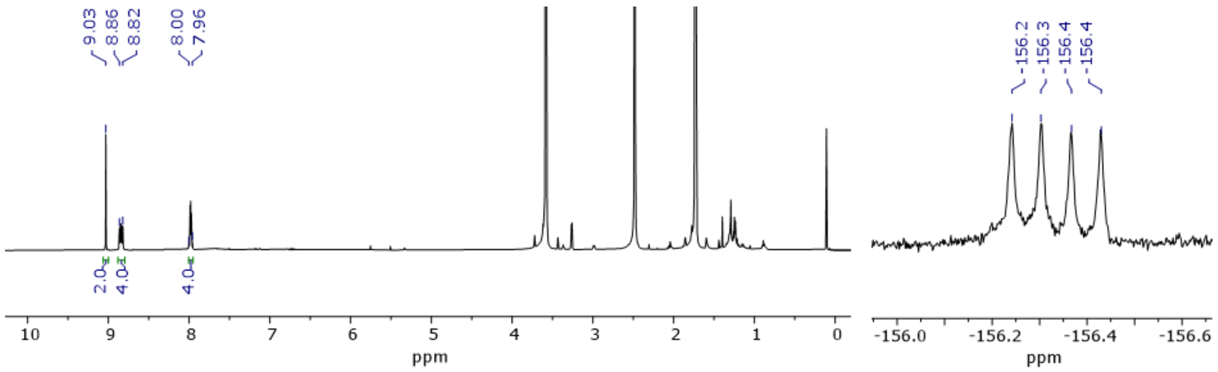

Figure S3.4. ^{1}H-NMR (THF-d_8, left) and ^{19}F-NMR (acetone-d_6, right) spectra of **Cl_2SubPc.**

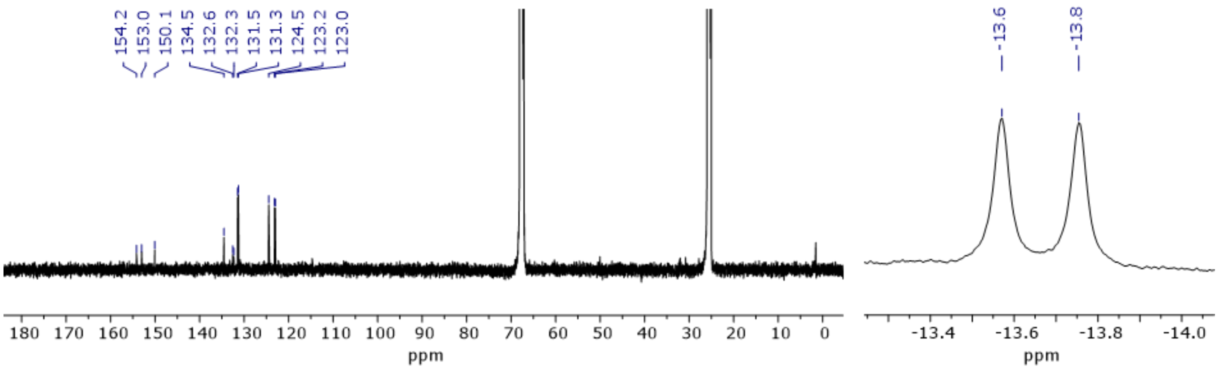

Figure S3.5. ^{13}C-NMR (THF-d_8, left) and ^{11}B-NMR (acetone-d_6, right) spectra of **Cl_2SubPc.**

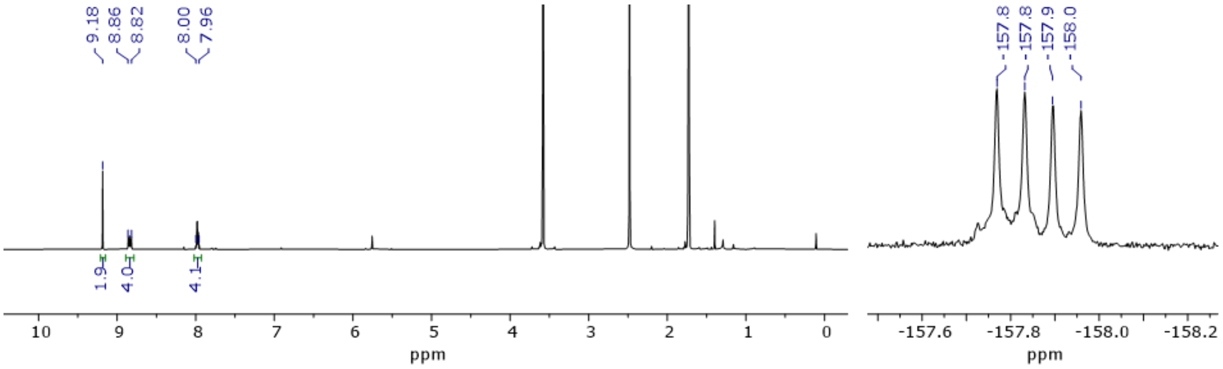

Figure S3.6. ^{1}H-NMR (left) and ^{19}F-NMR (right) spectra (THF-d$_8$) of **Br$_2$SubPc.**

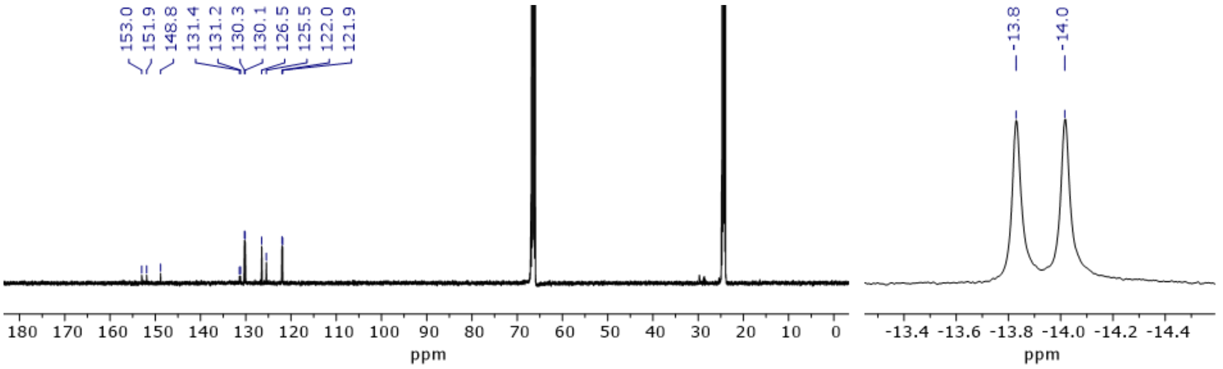

Figure S3.7. ^{13}C-NMR (left) and ^{11}B-NMR (right) spectra (THF-d$_8$) of **Br$_2$SubPc.**

4. Mass Spectra

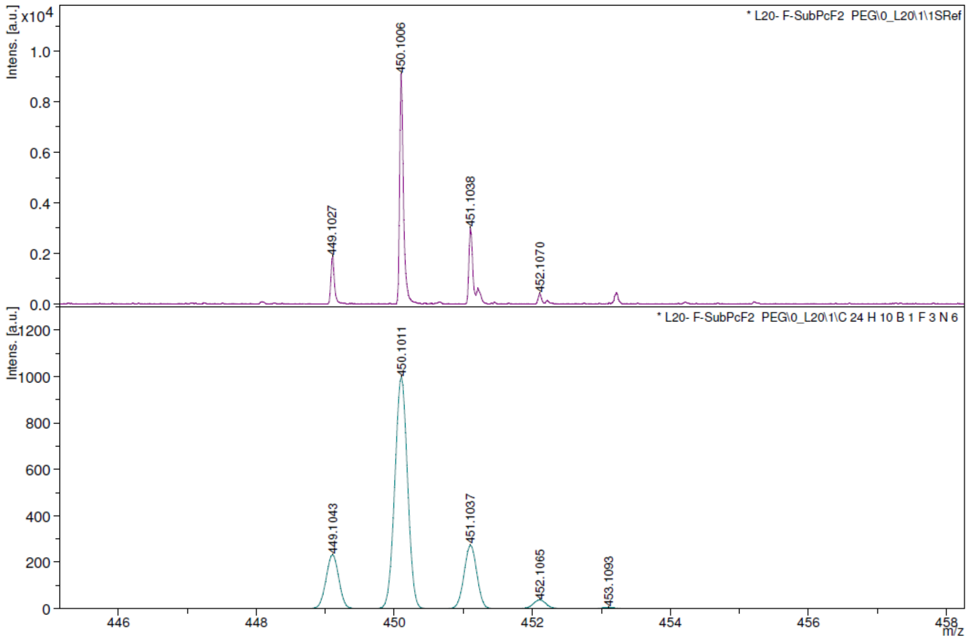

Figure S4.1. MALDI-TOF mass spectrum of **F_2SubPc.**

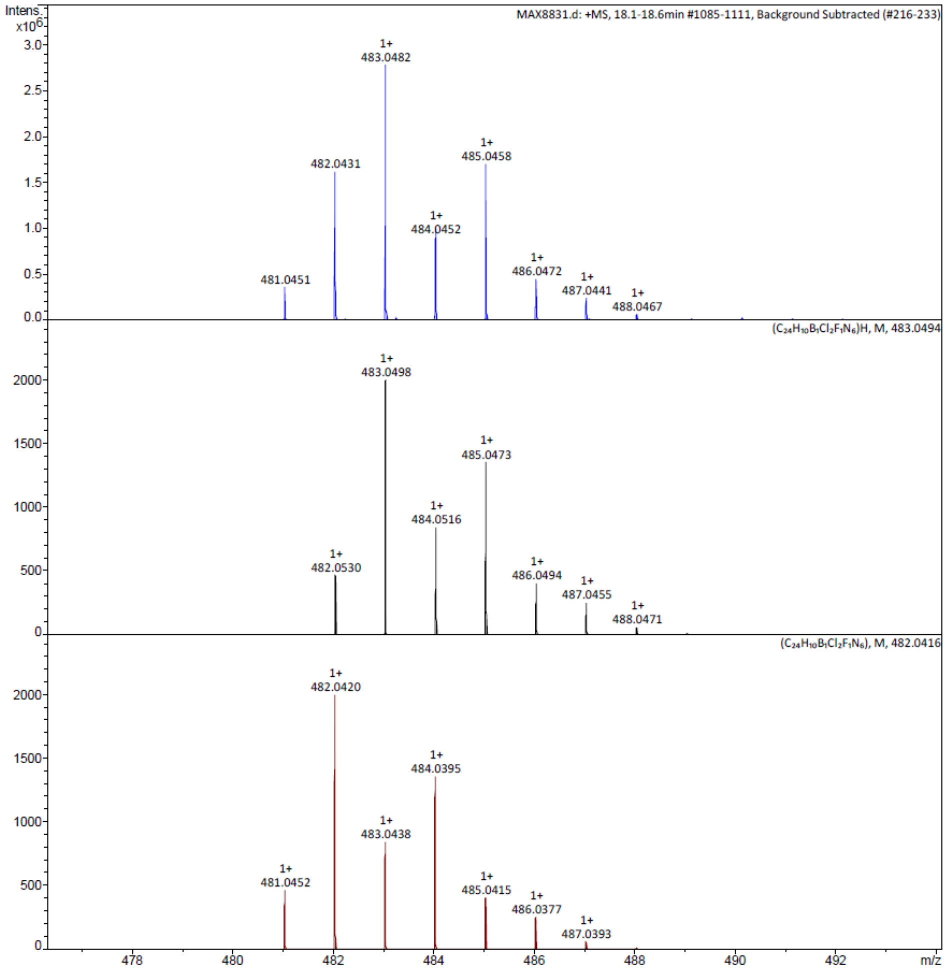

Figure S4.2. APCI-Q-TOF mass spectrum of **Cl_2SubPc.**

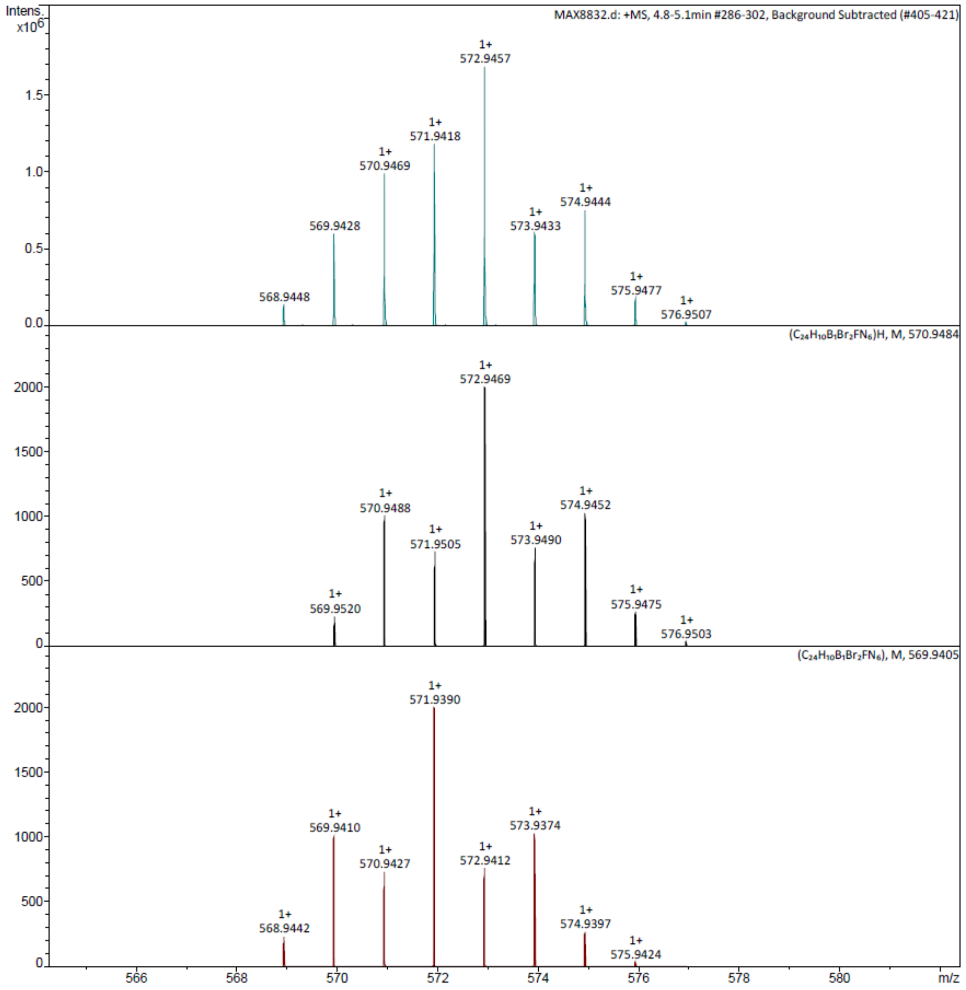

Figure S4.3. APCI-Q-TOF mass spectrum of **Br2SubPc.**

5. Complementary On-surface Studies

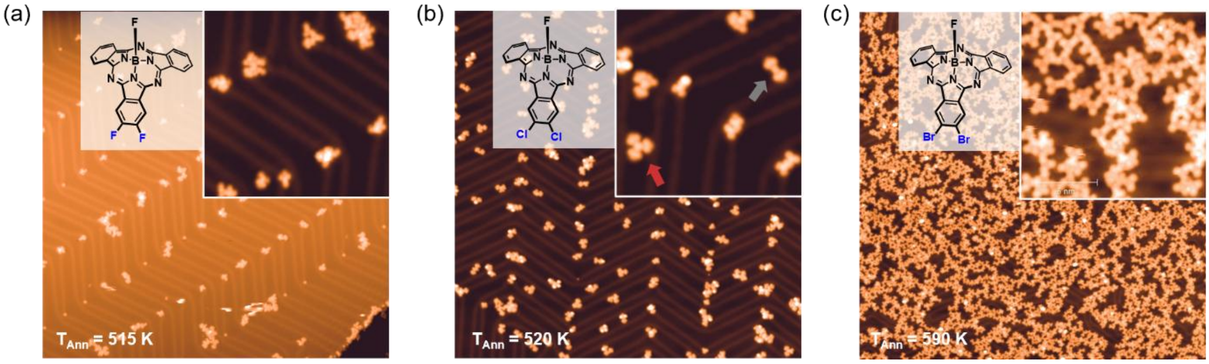

Figure S5.1. Elevated temperature arrangements of the three X_2SubPc species studied on the Au(111). At very high temperatures (above 500 K) all species reacted leading to covalent structures mostly irregular. In the case of F_2SubPc (a) no regular dimer or trimer was identified. (b) Cl_2SubPc exhibited covalent dimers (gray arrow) and trimers (red arrow) and notably Au atoms were observed to be attached at the *meso*-N atoms of the SubPc cores. (c) BrSubPc mainly presented covalent bonds between the benzene groups, which could be driven by the relative higher coverage used in this case.

STM image details: (a) V_B = 0.05 V, I_T = 10 pA, 108 x 108 nm^2, inset V_B = 0.01 V, I_T = 150 pA, 27 x27 nm^2;(b) V_B= 0.25 V, I_T = 10 pA, 108 x 108 nm^2, inset V_B = 0.05 V, I_T = 50 pA, 54 x 54 nm^2; (c) V_B = –0.3V, I_T = 50 pA, 162 x 162 nm^2, inset V_B = –0.01 V, I_T = 100 pA, 18.2 x 16.2 nm^2.

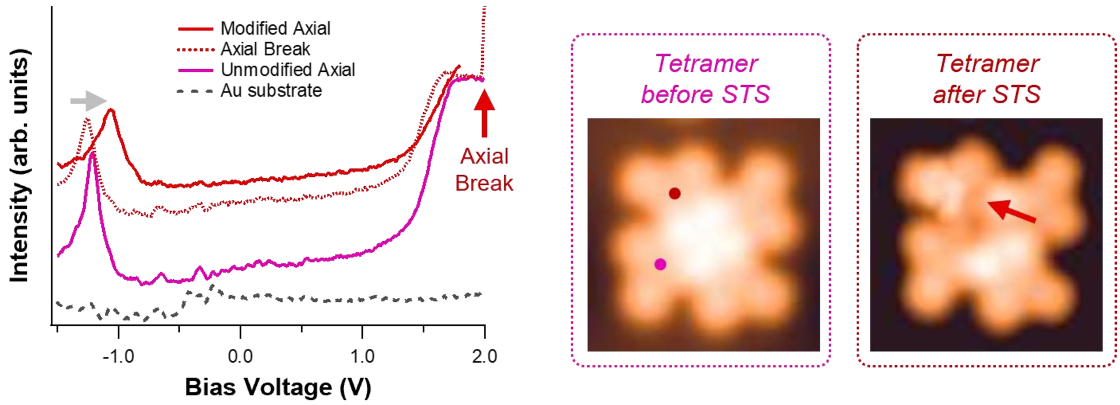

Figure S5.2. Modification of the central axial ligand while performing dI/dV curves. Performing conductance spectra on the axial ligand of one Br_2SubPc molecules conforming a tetramer often results in the alteration of this vacuum protruding bond. Particularly, such modification occurs while applying higher voltages than 1.95 V with currents over 1.2 nA. When this modification takes place, a sudden jump is observed on the spectrum at this bias (note this in the discontinuous red curve acquired while sweeping from lower to higher voltages). Repeating the dI/dV spectrum at this position (red dot in the left STM image) results in a conspicuous change in the spectrum, which differs strongly from the magenta curve showing the dI/dV curve from a pristine molecule at the same position. This modification of the axial ligand only affects this molecule, leaving unaffected the other three in the tetramer

(see right STM image). The discontinuous gray line dI/dV spectrum corresponds to a substrate and is shown for comparison.

Details of the STM images: V_B= –1.0V, I_T = 170pA, 3.8 x 3.8 nm^2. dI/dV spectra setpoint: –1.0 V, 200 pA with V_{RMS} = 9.5 mV, f = 817 Hz.

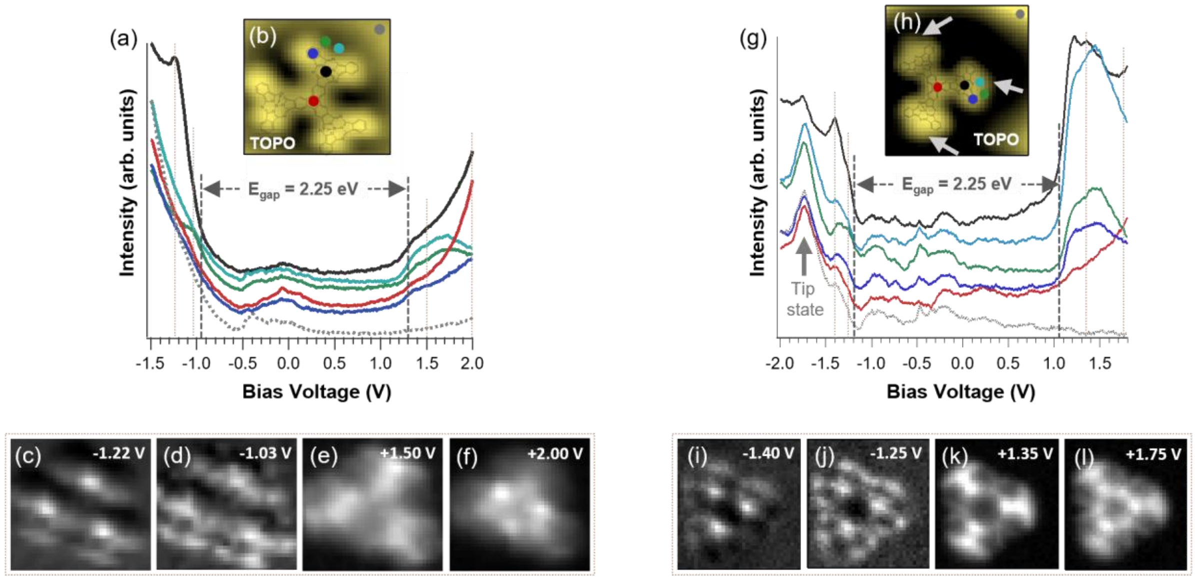

Figure S5.3. Electronic effect of Au adatoms attached to the covalent trimers at the meso-N atoms. The figure shows the electronic structure of two covalent trimers on Au(111): the left one generated from Br_2SubPc (a to f) and free of Au adatoms and the right one from Cl_2SubPc (g to l) with three Au adatoms attached, as indicated by arrows in panel (h). Both datasets have been extracted from dI/dV grids and shows the STS (a,g) at the selected positions indicated in the constant current topography (b, h), and four equivalent dI/dV isoenergetic maps (b-e and i-l). The resolution in the left dataset is better due to the functionalization of the tip. Note that the energies are shifted by 0.22 V to a higher value with respect to panels (c-e), also shown in Fig. 4 (q) to (s), which is caused by the absence of metal adatoms attached to the trimer edges.

Experimental details: Topo (a) V_B = –1.0 V, I_T = 200 pA, 2.9 x 2.7 nm^2; (h) V_B = 0.05 V, I_T = 10 pA, 4.2 x 4.4 nm^2. dI/dV grid (b) to (f) 20 x 19 points with setpoint at –1.0 V, 200 pA, V_{RMS} = 20 mV, f = 817 Hz; (g) to (l) 36 x 38 points with setpoint at 0.20 V, 40 pA, V_{RMS} = 9.5 mV, f = 817 Hz.

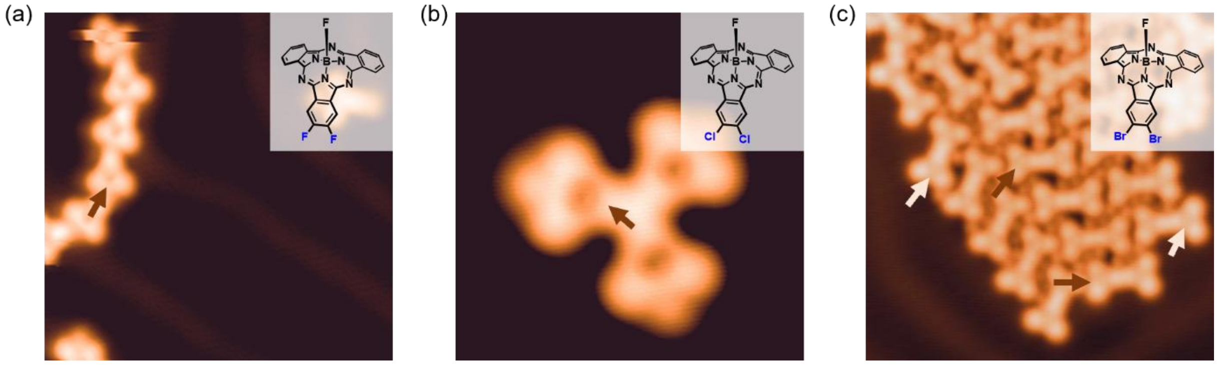

Figure S5.4. Close-up view of the axial ligand in the structures formed upon annealing X_2SubPc on Au(111) up to 475 K. Following the formation of covalent structures at elevated temperatures, the axial B─F bond remains intact across the derivatives of all three substrates. As illustrated in Figures (a─c), the distinct contrast observed at the center of the SubPc (indicated by arrows) results from the presence of the fluorine atom, which is retained after annealing.

STM image details: (a) V_B = 0.01 V, I_T = 10 pA, 10.8 x 10.8 nm^2; (b) V_B = –0.005 V, I_T = 180 pA, 4 x 4 nm^2; (c) V_B = –0.3 V, I_T = 100 pA, 11.9 x 11.9 nm^2.

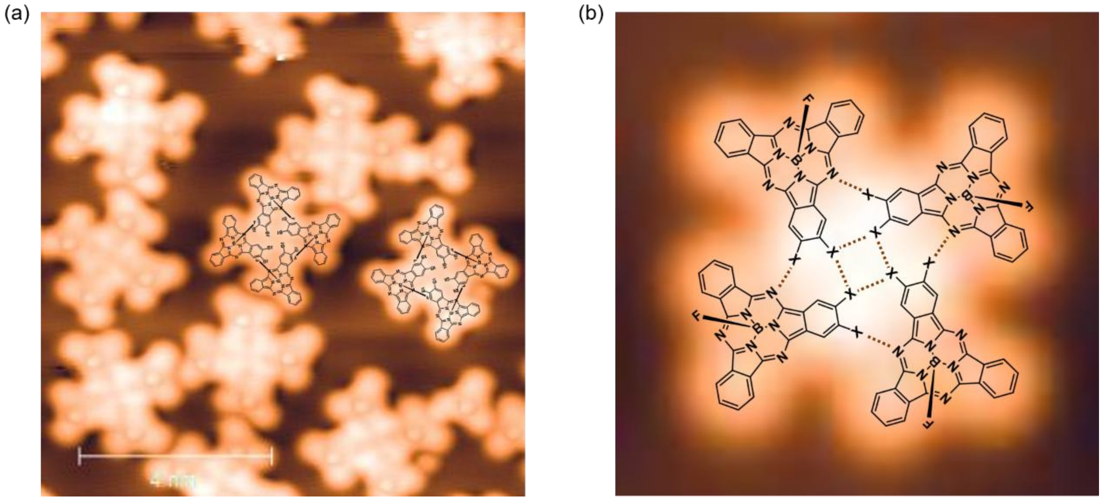

Figure S5.5. High-resolution image of several (a) and a single (b) tetramer formed upon deposition of Br_2SubPc at room temperature on Au(111). Schematic representations of the monomeric interactions have been superimposed to illustrate the molecular architecture of the tetramer and the dipole-dipole forces contributing to its stabilization. It is observed that the assembly is governed by halogen···N and halogen···halogen interactions. This structural model is applicable to the chiral supramolecular tetramers formed by both Cl_2SubPc and Br_2SubPc upon deposition at room temperature.

STM image details: (a) V_B = –1.0 V, I_T = 60 pA, 10 x 10 nm^2; (b) V_B = –1.05 V, I_T = 150 pA, 3.8 x 3.8 nm^2.

6. Complementary Computational Studies

Periodic density functional theory (DFT) calculations were carried out using the VASP package.[3] Core–valence interactions were described with the projector-augmented wave (PAW) method,[4] and the electronic wave functions were expanded in a plane-wave basis set with a kinetic-energy cutoff of 400 eV. Exchange–correlation effects were treated using the van der Waals density functional[5] in its rev-vdW-DF2 formulation,[6] which has been shown to accurately describe molecules adsorbed on coinage-metal surfaces.[7] The Au(111) surface was modeled by a three-layer slab. For adsorption of the covalent dimer, a p(12×9) surface unit cell was employed; for the covalent trimer, a p(12×12) surface unit cell was used; and for the organometallic dimer, a p(14×9) surface unit cell was adopted, together with a Γ-point only k-point sampling. Structures were optimized until residual forces on the atoms in the molecules and the outermost layers were smaller than 0.01 eV/Å. A vacuum spacing of 20 Å was applied between periodic Au slabs, corresponding to a separation exceeding 13 Å between the outermost atom of the adsorbed molecule and the bottom Au layer in the adjacent periodic image, which is sufficient to avoid spurious interactions between periodic replicas. The optimized lattice constant of Au used in this work was 4.122 Å. STM images were simulated within the Tersoff–Hamann approximation[8] as implemented by Lorente and Persson.[9]

For ESP maps, structures were optimized at DFT level, using the B3LYP functional and the 6-31G(d,p) calculation base.[10,11,12] these theoretical calculations were carried out using methods implemented in the Gaussian 16 package.[13]

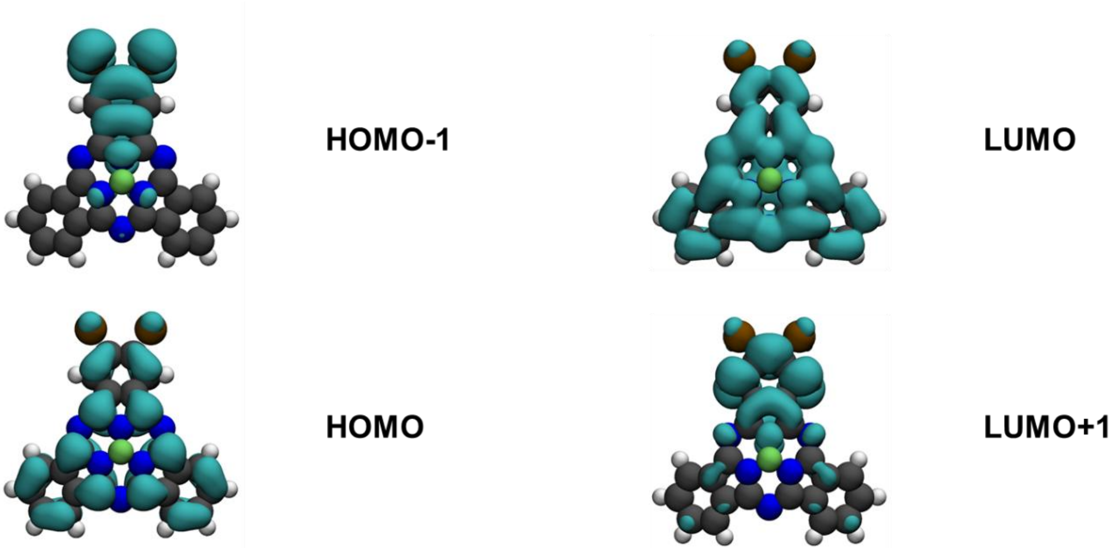

Figure S6.1. Simulated orbital densities of **Br_2SubPc** calculated in gas phase, going from HOMO-1 to LUMO+1.

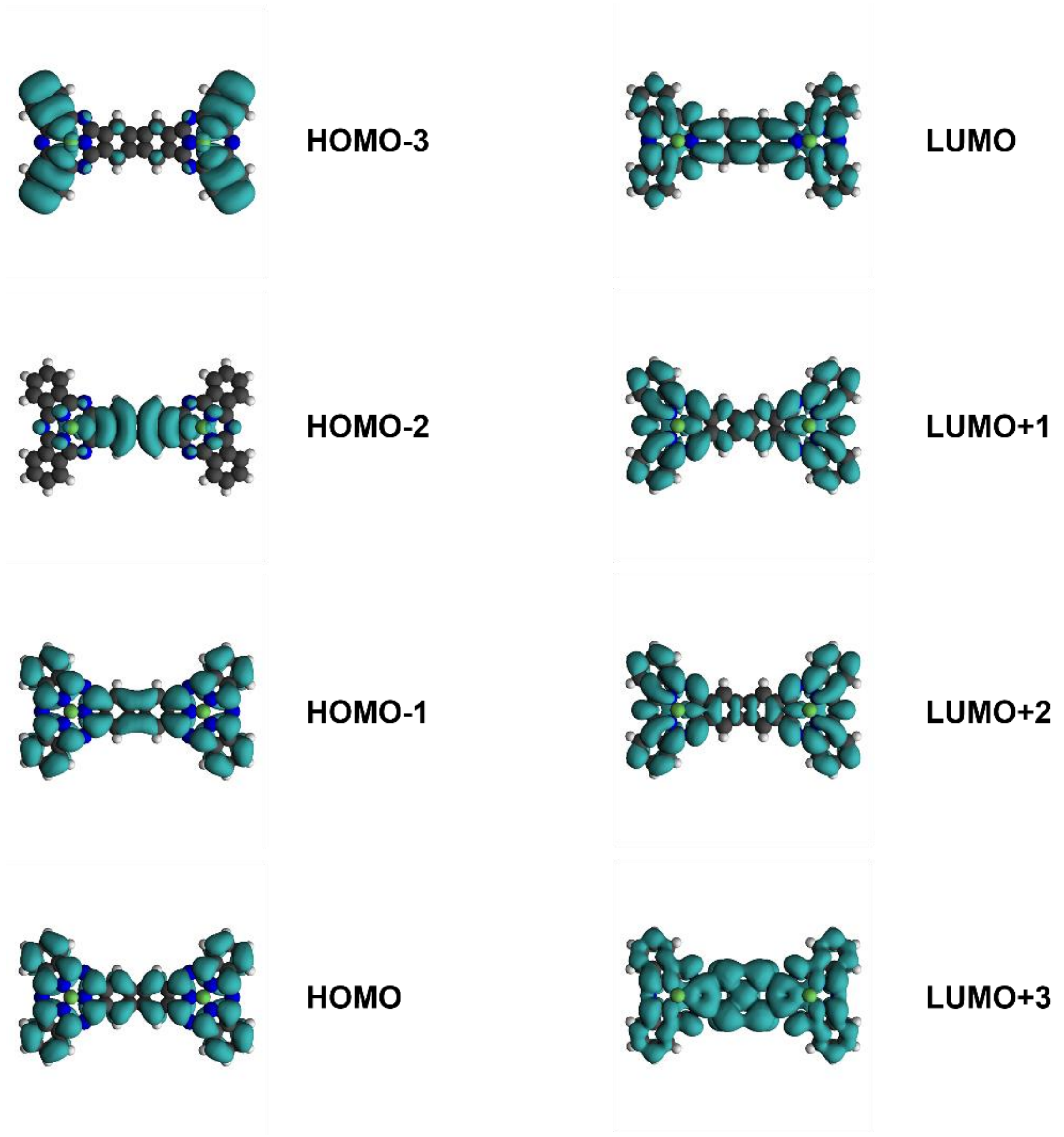

Figure S6.2. Simulated orbital densities of a covalent dimer calculated in gas phase, going from HOMO-3 to LUMO+3.

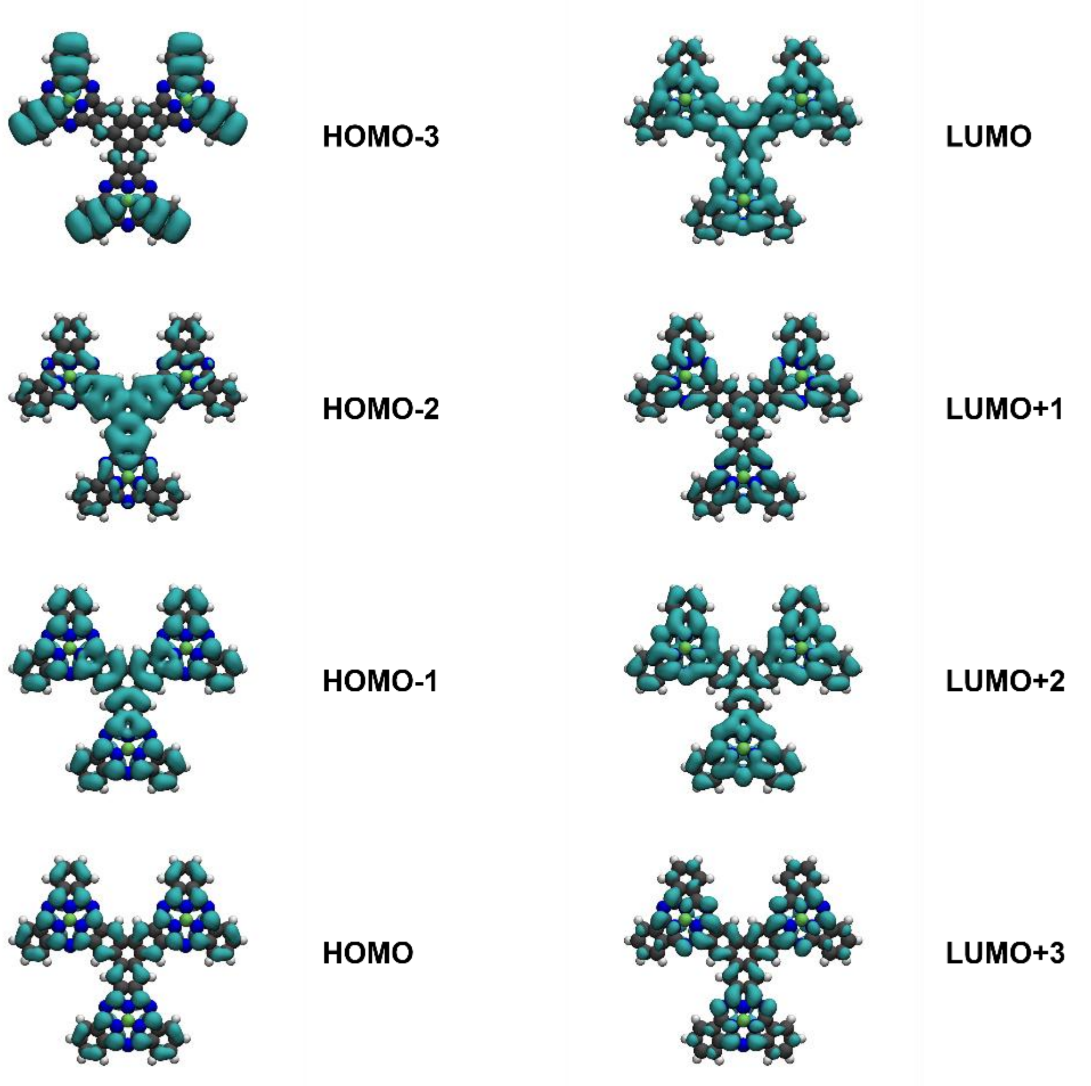

Figure S6.3. Simulated orbital densities of a covalent trimer calculated in gas phase, going from HOMO-3 to LUMO+3.

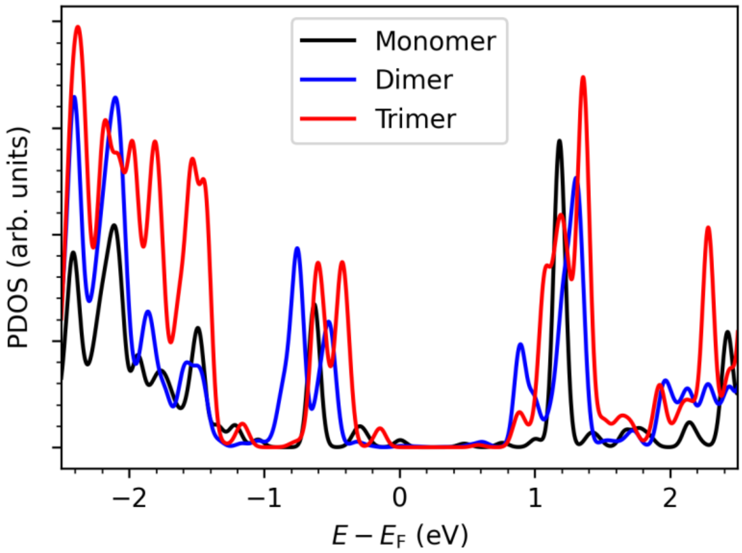

Figure S6.4. Simulated density of states projected onto the monomer, dimer, and trimer adsorbed on the Au(111) surface. For the LUMO, corresponding downward energy shifts of comparable magnitude are found for both the dimer and trimer. As a result, the HOMO–LUMO gap decreases systematically from monomer to dimer to trimer, with the trimer exhibiting the smallest gap and the monomer the largest.

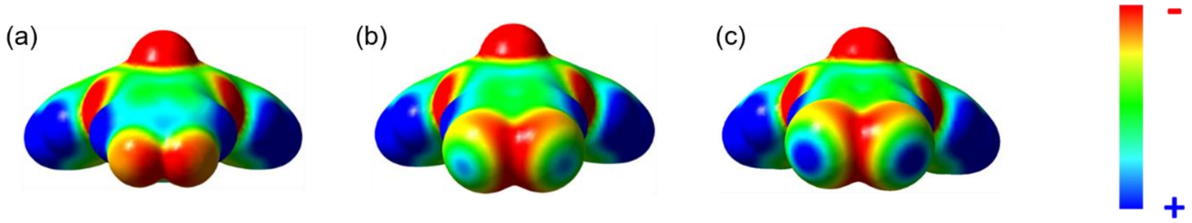

Figure S6.5. ESP maps of (a) F_2SubPc, (b) Cl_2SuPc, and (c) Br_2SubPc, calculated at B3LYP/6-31G(d,p) level in gas phase.

7. Supporting References

[1] Babij, N. R.; McCusker, E. O.; Whiteker, G. T.; Canturk, B.; Choy, N.; Creemer, L. C.; Amicis, C. V. D.; Hewlett, N. M.; Johnson, P. L.; Knobelsdorf, J. A.; Li, F.; Lorsbach, B. A.; Nugent, B. M.; Ryan, S. J.; Smith, M. R.; Yang, Q. NMR Chemical Shifts of Trace Impurities: Industrially Preferred Solvents Used in Process and Green Chemistry. *Org. Process Res. Dev.* **2016**, *20*, 661–667.

[2] Leznoff, C. C.; Li, Z.; Isago, H.; D'Ascanio, A. M.; Terekhov, D. S. Syntheses of Octaalkynylphthalocyanines from Halophthalonitriles. *J. Porphyr. Phthalocya.* **1999**, *03*, 406–416.

[3] Kresse, G.; Furthmüller, J. Efficient iterative schemes for ab initio total-energy calculations using a plane-wave basis set. *Phys. Rev. B* **1996**, *54*, 11169–11186.

[4] Blöchl, P. E. Projector augmented-wave method. *Phys. Rev. B* **1994**, *50*, 17953–17979.

[5] Dion, M.; Rydberg, H.; Schröder, E.; Langreth, D. C.; Lundqvist, B. I. Van der Waals Density Functional for General Geometries. *Phys. Rev. Lett.* **2004**, *92*, 246401.

[6] Hamada, I. van der Waals density functional made accurate. *Phys. Rev. B* **2014**, *89*, 121103.

[7] Björk, J.; Stafström, S. Adsorption of Large Hydrocarbons on Coinage Metals: A van der Waals Density Functional Study. *ChemPhysChem* **2014**, *15*, 2851–2858.

[8] Tersoff, J.; Hamann, D. R. Theory of the scanning tunneling microscope. *Phys. Rev. B* **1985**, *31*, 805–813.

[9] Lorente, N.; Persson, M. Theoretical aspects of tunneling-current-induced bond excitation and breaking at surfaces. *Faraday Discuss.* **2000**, *117*, 277–290.

[10] Lee, C.; Yang, W.; Parr, R. G. Development of the Colle-Salvetti correlation-energy formula into a functional of the electron density. *Phys. Rev. B* **1988**, *37*, 785–789.

[11] Becke, A. D. Density-functional thermochemistry. III. The role of exact exchange. *J. Chem. Phys.* **1993**, *98*, 5648–5652.

[12] Kohn, W.; Becke, A. D.; Parr, R. G. Density Functional Theory of Electronic Structure. *J. Phys. Chem.* **1996**, *100*, 12974–12980.

[13] Gaussian 16, Revision C.01; Frisch, M. J.; Trucks, G. W.; Schlegel, H. B.; Scuseria, G. E.; Robb, M. A.; Cheeseman, J. R.; Scalmani, G.; Barone, V.; Petersson, G. A.; Nakatsuji, H.; Li, X.; Caricato, M.; Marenich, A. V.; Bloino, J.; Janesko, B. G.; Gomperts, R.; Mennucci, B.; Hratchian, H. P.; Ortiz, J. V.; Izmaylov, A. F.; Sonnenberg, J. L.; Williams-Young, D.; Ding, F.; Lipparini, F.; Egidi, F.; Goings, J.; Peng, B.; Petrone, A.; Henderson, T.; Ranasinghe, D.; Zakrzewski, V. G.; Gao, J.; Rega, N.; Zheng, G.; Liang, W.; Hada, M.; Ehara, M.; Toyota, K.; Fukuda, R.; Hasegawa, J.; Ishida, M.; Nakajima, T.; Honda, Y.; Kitao, O.; Nakai, H.; Vreven, T.; Throssell, K.; Montgomery, J. A., Jr.; Peralta, J. E.; Ogliaro, F.; Bearpark, M. J.; Heyd, J. J.; Brothers, E. N.; Kudin, K. N.; Staroverov, V. N.; Keith, T. A.; Kobayashi, R.; Normand, J.; Raghavachari, K.; Rendell, A. P.; Burant, J. C.; Iyengar, S. S.; Tomasi, J.; Cossi, M.; Millam, J. M.; Klene, M.; Adamo, C.; Cammi, R.; Ochterski, J. W.; Martin, R. L.; Morokuma, K.; Farkas, O.; Foresman, J. B.; Fox, D. J. Gaussian, Inc., Wallingford CT, 2016.
